# Cyclophilin40 isomerase activity is regulated by a temperature-dependent allosteric interaction with Hsp90

**DOI:** 10.1042/BSR20150124

**Published:** 2015-10-19

**Authors:** Elizabeth A. Blackburn, Martin A. Wear, Vivian Landré, Vikram Narayan, Jia Ning, Burak Erman, Kathryn L. Ball, Malcolm D. Walkinshaw

**Affiliations:** * Centre for Translational and Chemical Biology, School of Biological Sciences, University of Edinburgh, Michael Swann Building, The King's Buildings, Mayfield Road, Edinburgh EH9 3JR, U.K.; † IGMM-Edinburgh Cancer Research Centre, University of Edinburgh, Crewe Road South, EH4 2XR, U.K.; ‡ Chemical and Biological Engineering Department, Koc University, Istanbul 34415, Turkey

**Keywords:** allostery, heat-shock protein 90, immunophilin, molecular dynamics, peptidyl-prolyl isomerase, tetratricopeptide (TPR) domain

## Abstract

Cyclophilin 40 (Cyp40) comprises an N-terminal cyclophilin domain with peptidyl-prolyl isomerase (PPIase) activity and a C-terminal tetratricopeptide repeat (TPR) domain that binds to the C-terminal–EEVD sequence common to both heat shock protein 70 (Hsp70) and Hsp90. We show in the present study that binding of peptides containing the MEEVD motif reduces the PPIase activity by ∼30%. CD and fluorescence assays show that the TPR domain is less stable than the cyclophilin domain and is stabilized by peptide binding. Isothermal titration calorimetry (ITC) shows that the affinity for the–MEEVD peptide is temperature sensitive in the physiological temperature range. Results from these biophysical studies fit with the MD simulations of the apo and holo (peptide-bound) structures which show a significant reduction in root mean square (RMS) fluctuation in both TPR and cyclophilin domains when–MEEVD is bound. The MD simulations of the apo-protein also highlight strong anti-correlated motions between residues around the PPIase-active site and a band of residues running across four of the seven helices in the TPR domain. Peptide binding leads to a distortion in the shape of the active site and a significant reduction in these strongly anti-correlated motions, providing an explanation for the allosteric effect of ligand binding and loss of PPIase activity. Together the experimental and MD results suggest that on heat shock, dissociation of Cyp40 from complexes mediated by the TPR domain leads to an increased pool of free Cyp40 capable of acting as an isomerase/chaperone in conditions of cellular stress.

## Abstract (precis)

Binding the C-terminus of heat shock protein 90 (Hsp 90) to the tetratricopeptide repeat (TPR) domain of cyclophilin 40 (Cyp40) allosterically changes the dynamics of the cyclophilin-active site and reduces peptidyl-prolyl isomerase (PPIase) activity.

## INTRODUCTION

Immunophilins are peptidyl-prolyl isomerases (PPIase) that include the structurally unrelated families of cyclophilins and FKBPs (FK506-binding proteins). Cyclophilin A (CypA), the archetypal cyclophilin, has a single PPIase domain of 165 amino acids and catalyses the inter-conversion between *cis* and *trans* forms of the peptidyl-prolyl bond (EC 5.2.1.8). There are 20 cyclophilin paralogues in the human genome which are found in diverse cellular compartments and form components of multiple cell signalling pathways and steroid receptor complexes [1–3]. The single domain prototypic cyclophilins show PPIase activity with *k***_cat_**/*K*_m_ values ranging from 5.5×10^5^ to 1.4×10^7^ M^−1^ s^−1^ [1]. A likely consequence of this enzymatic activity is their ability to catalyse the rate of protein folding in the cell. In various *in vitro* assays, protein folding speeds up from seconds to milliseconds [4]. NMR relaxation experiments have been used to show that intrinsic CypA motions occur with frequencies that are similar to the catalytic turnover of peptide substrates measured at between 12000 and 13000 s^−1^ [5,6]. Cyclophilins are known to associate with a wide variety of protein partners and are present in large protein complexes as co-chaperones [7]. Whether the presence of a PPIase domain confers molecular recognition, isomerase or chaperone activity is not fully understood but is likely to be dependent on both the protein partner and the nature of the additional domains present in the multi-domain cyclophilins. A ligand binding to an accessory domain has been shown to influence PPIase activity in a number of multi-domain PPIases. FKBP38 contains a PPIase domain and a C-terminal TPR domain that binds heat shock protein 90 (Hsp90). The PPIase activity of FKBP38 is stimulated by the binding of Ca^2+^/calmodulin to the unusual N-terminal extension of the PPIase domain. In contrast, PPIase activity is reduced when Hsp90 binds to the TPR domain [8]. Cyclophilin 33 (Cyp33) contains an RNA-binding domain; PPIase activity is increased by mRNA binding [9]. However, the PPIase activity of *At*Cyp59 (Arabidopsis thaliana cyclophilin 59) is inhibited by RNA binding [10]. Allosteric inter-domain communication influences the PPIase activity of Pin1 (peptidyl-prolyl cis-trans isomerase NIMA-interacting 1) [11,12].

Another near-universal property of the cyclophilins is the tight binding to, and inhibition by, the immunosuppressant peptide drug cyclosporin A (CsA). This cyclic undecapeptide binds in the active site of most cyclophilin paralogues with *K*_d_ values ranging from very low nanomolar to micromolar [13].

**Figure 1:**
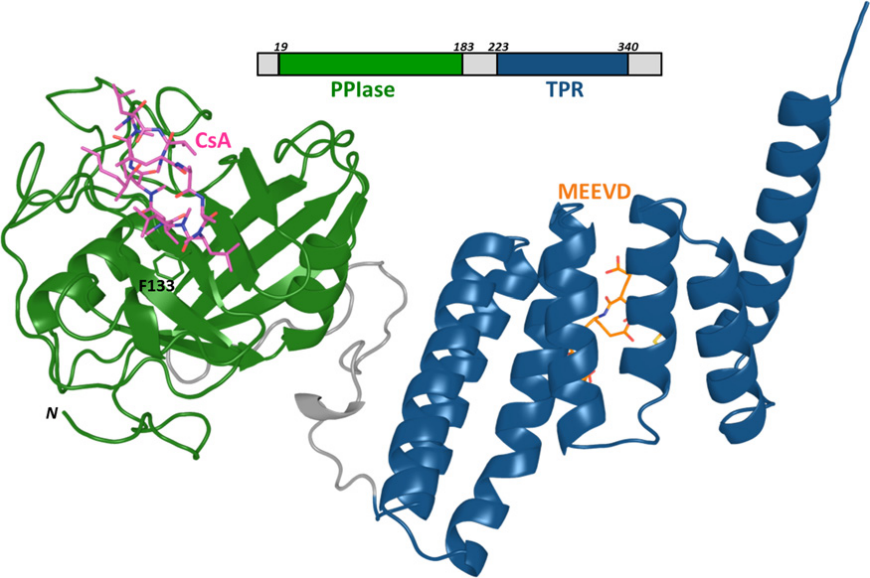
Cyp40 architecture Cyp40 has a single N-terminal cyclophilin domain (green) connected by a 30-amino acid linker to a TPR domain (blue) composed of three tandem TPRs. Phe^133^ forms the base of the active site. Cyp40 (1IHG.pdb) modelled with CsA (magenta), based on an alignment of CypA in complex with CsA (1CWA.pdb) and MEEVD peptide (orange), based on an alignment of CHIP in complex with peptide (2C2L.pdb).

In the present paper, we investigate the allosteric regulation of the enzyme activity of cyclophilin 40 (known as Cyp40 and more confusingly as PPID). Cyp40 has high catalytic efficiency (*k*_cat_/*K*_m_=1.3×10^7^ M^−1^ s^−1^), similar to that of CypA (*k*_cat_/*K*_m_=1.4×10^7^ M^−1^ s^−1^). The affinity for CsA is 61 nM, within the range of the prototypic cyclophilins which have *K*_d_ between 10 and 100 nM [13]. Cyp40, in common with seven of the FKBPs, has a C-terminal TPR domain made up of three helix-turn-helix tetratricopeptide repeat (TPR) motifs. The single N-terminal cyclophilin domain of Cyp40 is connected by a 30 amino acid linker to the TPR domain (Figure 1) [14]. Cyp40 is frequently grouped with the well characterized ‘large immunophilins’ FKBP51 and 52, due to their similar size and associated biological roles as part of steroid receptors. The TPR domains of FKBP51 and FKBP52 share 25% and 28% identity respectively with the TPR domain of Cyp40. The immunophilin TPR proteins form a sub-set of a bigger group of proteins containing a TPR domain which bind competitively to Hsp90 or Hsp70 and act as co-chaperones [15]. Low sequence identities between TPR domains is characteristic of the fold and masks good conservation between specific residues in the immunophilin co-chaperone TPRs that make key interactions with Hsps (Supplementary Figure S1). There is a 75-fold increase in Cyp40 mRNA levels on heat shock but also increased levels of degradation leading to similar protein levels. One could imply from this that Cyp40 has an important cellular role in times of heat stress [16].

Binding of the TPR domain to the Hsp is via their C-terminal amino acids; Hsp90 has the terminal sequence MEEVD-COOH and Hsp70 has the C-terminal sequence IEEVD-COOH [17]. Cyp40 has a preference for Hsp90 and has been found associated with Hsp90 as a binary complex and as a component of the mature unactivated AR (androgen receptor), ER (estrogen receptor), PR (progesterone receptor) and steroid receptor complex. It also acts as a facilitator of membrane transfer for clostridial ADP-ribosyltransferases [18–22]. Bovine Cyp40 (*b*Cyp40) has been crystallized as an apo protein with the TPR domain in two conformations. In 1IHG.pdb the TPR domain adopts the typical TPR fold and 1IIG.pdb where two of the TPR helices have straightened out to form one extended helix [14]. To date, X-ray structures of eight distinct TPR domains have been solved from five human proteins bound to Hsp90 or Hsp70 C-terminal peptides [RPAP3:4CGV.pdb-TPR1(SRMEEVD) and 4CGW.pdb-TPR2(SRMEEVD); CHIP:-4KBQ.pdb-TPR(Hsp70 C-terminal and lid fragments); AIP:4AI-F.pdb-TPR(SRMEEVD); HOP:1ELW.pdb-TPR1(GPTIEEVD), 3ESK.pdb-TPR2A(GPTIEEVD) and 1ELR.pdb-TPR2B(ME-EVD); FKBP52:1QZ2.pdb-TPR(MEEVD)]. All show a peptide lying in a groove between helix 1 and helix 5 and the C-terminus bound in a ‘carboxy clamp’ pocket (Figure 1). This proposed binding mode is also supported by a study of site point mutations of Cyp40 which identified key interacting residues [23].

We show in the present paper that binding of ligand to the TPR domain has an allosteric effect on the PPIase activity. The classic Monod—Wyman–Changeux model of allostery describes a conformational switch between two well-defined low-energy forms, but there is growing evidence to show that allostery may be understood in terms of an alteration of protein flexibility or reshaping of the energy landscape upon effector binding [24]. Biophysical measurements using fluorescence, CD spectroscopy and thermal denaturation spectroscopy have all been used to monitor the melting behaviour of Cyp40 in the presence and absence of peptide ligand. These measurements are combined with MD and enzymatic results to suggest that the observed allosteric behaviour is through regulation of protein flexibility and, in particular, by changes in correlated motions which can be adjusted by ligand binding.

## EXPERIMENTAL

### Protein production

Cyp40 (pDEST14-6H-TEV-*h*Cyp40) and CypA were overexpressed in *E.coli* [BL21 star™ (DE3), Life Technologies] essentially as described for CypA by Wear et al. [25]. In summary, proteins were purified to homogeneity by ion-metal affinity chromatography (1 ml, HiTrap IMAC,ff; GE Healthcare Life Sciences) followed by gel filtration (Superdex 200 10/300 GL). Purity was assessed to be greater than 95% by SDS/PAGE. The hydrodynamic properties of Cyp40 and CypA were assessed by dynamic light scattering; they showed no measurable aggregation and were consistent with those of monomeric protein when compared with X-ray crystallographic co-ordinates (Zetasizer, Malvern Instruments; Hydropro10) [26].

### Peptidyl-prolyl isomerase activity

PPIase activity of Cyp40 (10 nM) was compared for the unliganded (apo) form and in the presence of 250 μM XXXXXMEEVD peptide (holo) at 10°C with the substrate Suc-A-A-P-F-p-NA (Sigma); 50 mM Hepes, pH 8; 100 mM sodium chloride; 1 mM DTT determined as previously described [27]. using the method of Kofron (Jasco V-550 UV/vis spectrometer) [28].

### CD spectroscopy

The far UV CD spectrum of 0.75 μM Cyp40 was recorded at 20 nm min^−1^; data pitch 0.1 nm; response time 1 s between 185 and 285 nm in a 0.1 cm path-length quartz cuvette between 10°C and 60°C at 10°C intervals (JASCO-810 spectrometer). The protein was exchanged into 10 mM sodium phosphate, pH 8; 150 mM sodium fluoride prior to analysis (HiTrap desalt column, GE). Spectra were corrected by subtracting a buffer baseline recorded at each temperature, spectra were recorded in triplicate. Secondary structure was estimated using the Dichroweb CD secondary structure analysis server using the methods CONTIN, SELCON3 and CDSSTR and data set SP175; mean results are presented with S.E.M [29–34].

### Intrinsic tryptophan fluorescence

Steady-state fluorescent emission was recorded for 1 μM Cyp40 in the apo form and after the addition of 250 μM SRMEEVD (Hsp90 peptide) between 15°C and 45°C at 5°C intervals in 50 mM Hepes, pH 8; 100 mM sodium chloride; 1 mM DTT (Fluoromax-3 spectrometer, Horiba). Tryptophan was selectively excited at 295 nm (5 nm band pass excitation and emission) and emission spectra recorded from 310 to 400 nm, 1 nm interval; integration time 1 s; scans were repeated in triplicate, mean spectra are presented. Protein scans were blanked by subtracting a spectrum of a similar solution without protein at each temperature.

### Thermal denaturation fluorescence

Thermal denaturation fluorescence (TDF) melting curves were recorded for 2 μM Cyp40 in the apo form and in the presence of 4 μM CsA and 250 μM SRMEEVD peptide alone or together; 50 mM Hepes, pH8; 100 mM sodium chloride; 1 mM DTT. Fluorescence intensity was measured in relative fluorescence units (RFU) using excitation/emission wavelengths of 485 nm/575 nm between 25°C and 70°C, interval 0.5°C, 30 s hold at each temperature as previously described (IQcycler5, Bio-Rad) [35]. Data were collected in triplicated, mean thermograms are presented.

### Isothermal titration calorimetry

Isothermal titration calorimetry (ITC) experiments were carried out to determine the affinity of Cyp40 for Hsp90 peptide over the temperature range 10°C–30°C at 5°C intervals (Auto-iTC200 microcalorimeter; Malvern Instruments). Cyp40 was exchanged into the experimental buffer; 50 mM Hepes, pH 8; 100 mM sodium chloride; 1 mM DTT (HiTrap desalt column, GE Healthcare Life Sciences) immediately before the experiment. SRMEEVD (500 μM) was titrated into 7.7 μM Cyp40 in 15 × 2.5 μl aliquots. Data were analysed using non-linear regression in the MicroCal ORIGIN software package assuming a 1:1 binding model.

### MD simulations

All the MD simulations were carried out in explicit solvent (water) using NAMD 2.9 with CHARMM27 force field [36]. All simulations were performed at constant temperature (310 K) and pressure [1.01325 bar (1 bar=100 kPa)] in a periodic water box with a 20 Å (1 Å=0.1 nm) cushion. Ions were added to neutralize the system. Non-bonded and electrostatic forces were evaluated each time step. No rigid bonds were used. The cut-off distance was set to 12 Å to evaluate non-bonded interactions. The Particle Ewald sum was used in calculating long-range forces in the periodic systems, thereby minimizing the error introduced by truncation owing to the cut-off distance. Integration time step was set to 1 fs and structure was recorded at 1000-step (1 ps in MD). The system was subjected to three minimization–equilibration cycles prior to MD calculations. The first one was applied to relax the water and the last two were applied to find a local energy minimum of the whole system. All minimization cycles included 20000 energy minimization steps to relieve close intermolecular contacts and geometric strain. Simulations were carried out for 100 ns.

## RESULTS

### Binding MEEVD to the TPR domain of Cyp40 allosterically influences PPIase activity

**Figure 2:**
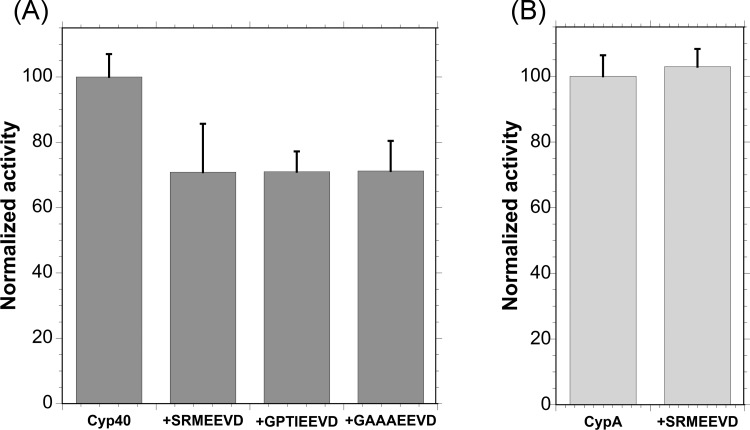
Binding MEEVD to the TPR domain of Cyp40 allosterically inhibits PPIase activity (**A**) Addition of the peptide containing the Hsp C-terminal sequence,–EEVD, to Cyp40 reduced PPIase activity by 30%. (**B**) A control experiment showing that the Hsp90 peptide does not affect PPIase activity by binding competitively to the Cyp40-active site and competing with the PPIase peptide substrate (AAPF-p-NA). Measurement was carried out using the single domain CypA. Mean of five repeats and S.E.M. corrected for thermal turnover.

Figure 2(A) shows the PPIase activity of Cyp40 (10 nM) measured in the presence and absence of the Hsp C-terminal peptides SRMEEVD [Hsp90 wt (wild-type)], GPTIEEVD (Hsp70 wt) and GAAAEEVD (Hsp70 mutant). In each case, addition of the peptide (250 μM) resulted in a reduction in the PPIase activity of over 30%. In order to show that the Hsp peptides were not affecting the assay by binding competitively to the Cyp40-active site and competing with the PPIase peptide substrate peptide (AAPF-p-NA) a control measurement was carried out using the single-domain CypA, no reduction in activity was observed (Figure 2B). The Hsp peptides bind to Cyp40 with *K*_d_ values of ∼5 μM (10°C; ITC data for SRMEEVD) and at the concentration used in this assay an estimated over 98% of Cyp40 would be bound [37]. The similar reduction in enzyme activity for all three peptides (XXXXEEVD) suggests that it is the binding of–EEVD to the dicarboxy clamp region of the TPR domain that causes this inhibitory effect.

### CD melting experiments

**Figure 3:**
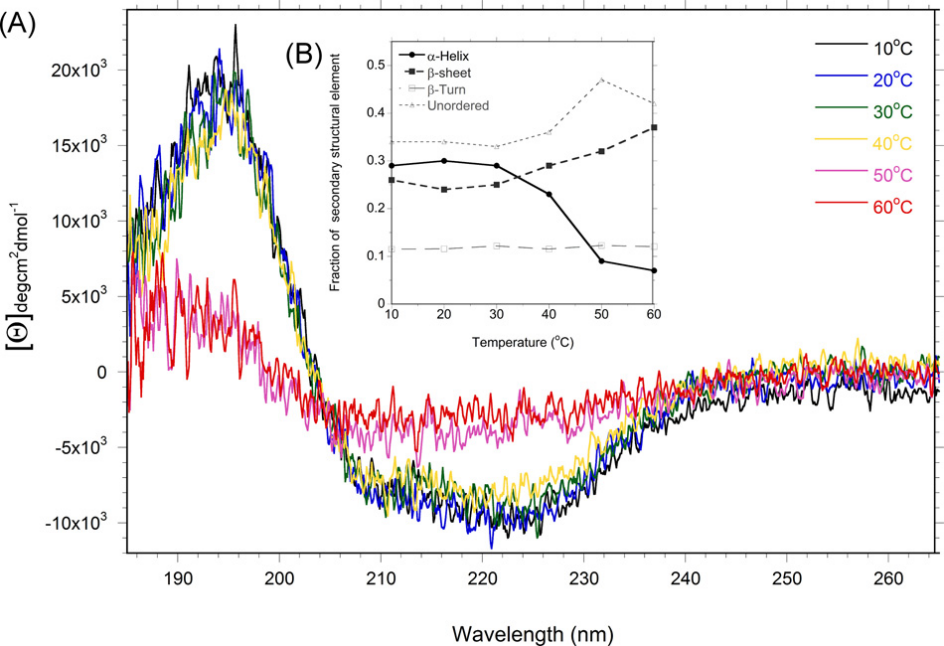
Melting of Cyp40 monitored by CD spectroscopy (**A**) Changes to the CD spectra of Cyp40 from 10°C to 60°C indicate that melting starts between 30°C (green) and 40°C (yellow). Inset (**B**) Analysis of the secondary structural elements, as a fraction of the total protein.

The change in secondary structure as a function of increasing temperature was monitored using CD spectroscopy (Figure 3A). The percentage of helix, sheet and random coil was estimated for each temperature (Figure 3B). Changes to secondary structure are first seen above 30°C as a reduced signal in the α-helical region of the spectrum. The cyclophilin domain of Cyp40 is largely β-sheet and the TPR domain largely α-helical (Figure 1).

### Intrinsic tryptophan fluorescence monitors flexibility in the TPR domain

**Figure 4:**
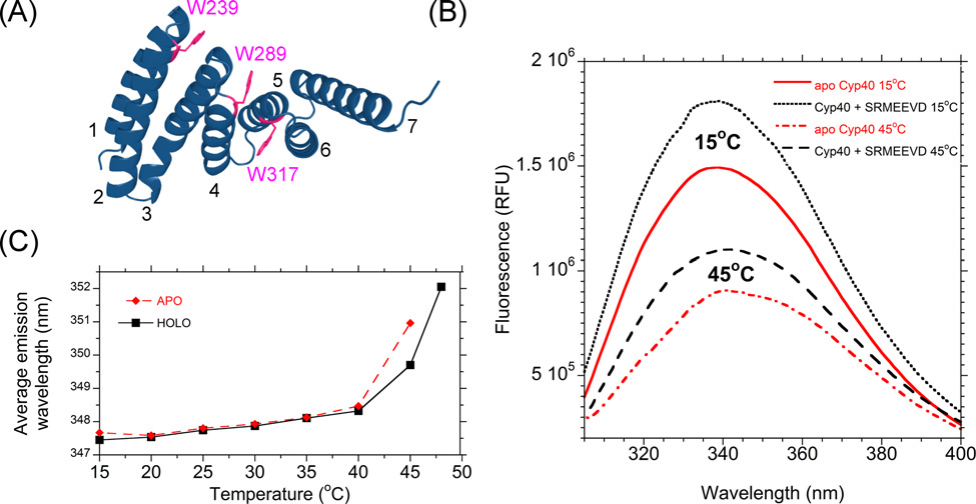
Melting of the Cyp40 TPR domain monitored by tryptophan fluorescence (**A**) The three tryptophan residues of *h*Cyp40 are located in the TPR domain at key interfaces between the three tandem TPRs. Helices are labelled 1–6. (**B**) Steady state-fluorescence is strongly influenced by both temperature and ligand binding. (**C**) Average emission wavelength increases with temperature and is decreased upon ligand binding.

Human Cyp40 is unusual in the cyclophilin family in that there are no tryptophan residues in the PPIase domain or linker region. There are three tryptophan residues in the TPR domain (Figure 4A). These residues are positioned at the interfaces of the TPRs where the motif fold back on each other and make hydrophobic contacts. Tryptophan can therefore be considered a good intrinsic probe of TPR dynamics as increased flexibility or unfolding of the TPR domains would alter chemical environment [38]. Trp^239^ forms an interface between the hairpin loop of helix 1 and 2 and helix 3; it makes hydrophobic contacts with Phe^234^, Gln^237^, Lys^285^ and Met^286^ and shows little solvent exposure. The crystal structure of *b*Cyp40 has high B-factors at the hinge between at the opposite ends of helix 2 and 3 (Glu^261^, Asp^262^, Ala^263^). Movement, perpendicular to the plane of the indole ring, at this hinge would have a direct effect on the environment of Trp^239^ and lead to exposure of the face of the indole group to solvent and the increased possibility of rotation about Cβ γ. Trp^289^ forms contacts between helices 3 and 4 and helix 5 and Trp^317^ in buried in the groove formed by helices 4, 5 and 6.

Typically solvent-exposed tryptophan residues fluoresce at a longer wavelength and have a smaller quantum yield than buried residues [39]; thus fluorescent emission provides a time-averaged reporter of the degree of buriedness of the indole rings. The changes in fluorescence signal between 20°C and 40°C (drop in signal and red shift from 348.3 to 351 nm) are consistent with a steady increase in solvent exposure. At 40°C, there is a marked change and the average emission wavelength red shifts from 348.3 to 351 nm (Figures 4B and 4C).

Addition of Hsp90 peptide causes an enhancement in fluorescence at all temperatures and is consistent with the tryptophan side chains being in a less solvent-exposed environment, though the red shift observed on melting is similar to the unliganded protein (Figures 4B and 4C).

### Thermal denaturation fluorescence melting experiments confirm that the TPR domain is mobile at physiological temperatures

**Figure 5:**
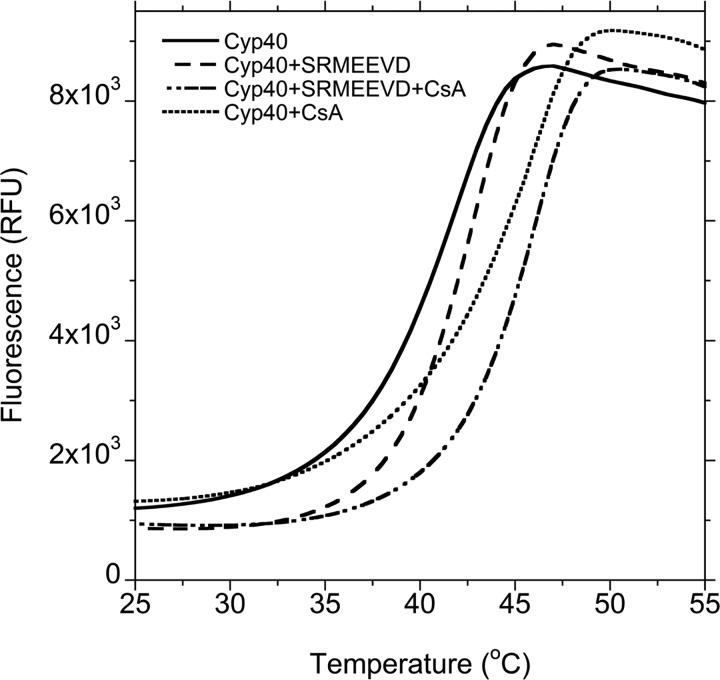
TDF assay shows Hsp90 peptide stabilizes the TPR domain Apo Cyp40 has a *T*_m_=42°C but starts to melt at 30°C (solid line). Addition of Hsp90 peptide–MEEVD delays onset of melting by ∼5°C; however, *T*_m_ only increases by ∼1°C (dashed line). Comparison of the shapes of these two melting curves suggests that the thermally unstable TPR domain is being stabilized by Hsp90 peptide at temperatures below those where the cyclophilin domain becomes destabilized. Addition of the tight binding CsA ligand (PPIase domain) raises the *T*_m_ by 4°C upto 46°C (dot line). The shape of the melting curve for addition of the MEEVD peptide along with CsA again suggests a delayed melting of the TPR (dash/dot line).

The increase in fluorescence measured in the TDF assay results from a dye probe binding to exposed hydrophobic residues as the protein melts and unfolds. The melting curves shown in Figure 5 show that apo Cyp40 has a *T*_m_=42°C (mid-point melting temperature) but starts to melt at 30°C (solid line). Addition of Hsp90 peptide -MEEVD delays onset of melting by ∼5°C, however *T*_m_ only increases by ∼1°C (dashed line). Comparison of the shapes of these two melting curves suggests that the TPR domain and PPIase domain do not start to melt at the same temperature. Addition of Hsp90 peptide stabilizes the TPR domain and influences the shape of the melting curve at temperatures below those where the cyclophilin domain is significantly destabilized. Addition of the tight-binding CsA ligand (PPIase domain) raises the *T*_m_ by 4°C upto 46°C. The shape of the melting curve for addition of the MEEVD peptide along with CsA again suggests a delay in melting of the TPR domain but this has little effect on the final *T*_m_ of the protein which remains at 46°C.

### ITC: the thermodynamics of binding of Cyp40 to Hsp90 shows remarkable temperature sensitivity in the physiological temperature range

**Figure 6:**
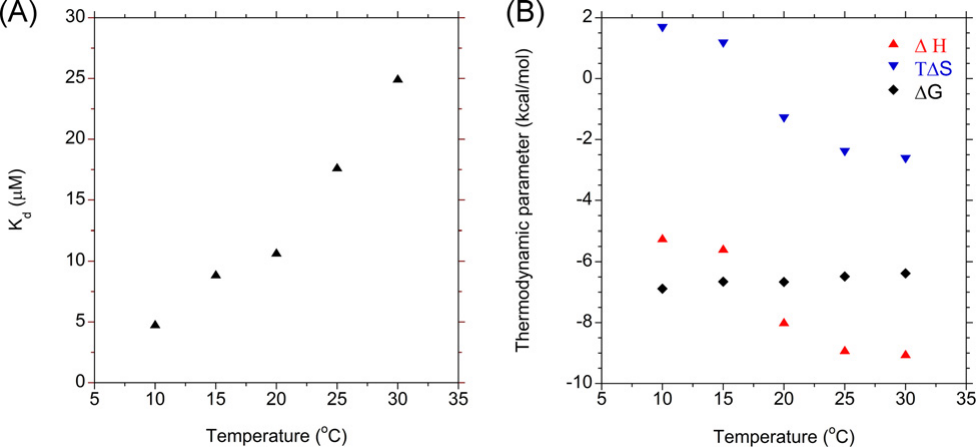
The temperature dependence of the affinity of Hsp90 peptide for Cyp40 (**A**) The affinity of Hsp90 peptide for *h*Cyp40 as a function of temperature. (**B**) Thermodynamic profile of peptide binding between 10°C and 30°C.

ITC data measured by titrating peptide and CypA at different temperatures shows that the there is a strong dependence on temperature for all of the thermodynamic parameters (Figures 6A and 6B). At 10°C the *K*_d_ for Cyp40 and the MEEVD peptide is ∼5 μM which rises to a value of ∼25 μM over which was measured at the highest attainable temperature of 30°C. The graph of changes in Δ*H* and Δ*S* with temperature shows a point of inflection between 25°C and 30°C suggesting, in line with data from CD and fluorescence, this corresponds with the onset of melting of the TPR (Figure 4). The gradient of Δ*H* against temperature also shows that Δ*C*_p_ is not constant between 25°C and 30°C. This may be explained by the likely change in water structure as the helices of the TPR domain become more flexible and begin to unfold.

### MD simulations

MD simulations were performed on apo Cyp40 (with no peptide ligand bound) using the X-ray structure 1IHG.pdb as the starting model. A second MD simulation was carried out on holo-Cyp40 with the MEEVD peptide docked in the binding groove of the TPR domain of Cyp40. The X-ray structure of the E3 ligase CHIP (PDB code 2C2L) has a peptide with an MEEVD motif bound to a TPR domain that is structurally homologous to the TPR domain of Cyp40 (choice of peptide structure is described in the Supplementary Material). A model of holo Cyp40 (bound to the peptide MEEVD) was generated by overlaying the two structures. The RMS fit for 90 corresponding atoms from the two structures is 1.1 Å. MD simulations were calculated for 100 ns using this docked structure as the starting model for holo Cyp40.

Average fluctuations (mean square displacement values) for each residue were calculated from the 100 ns simulations. The mean square displacement values for each atom <(Δ*R*_i_)^2^> are related to the temperature factor (*B*_i_) by:

$$<{\left(\Delta R_{i}\right)}^{2}=\left(3/8\pi^{2}\right)B_{i}$$

**Figure 7:**
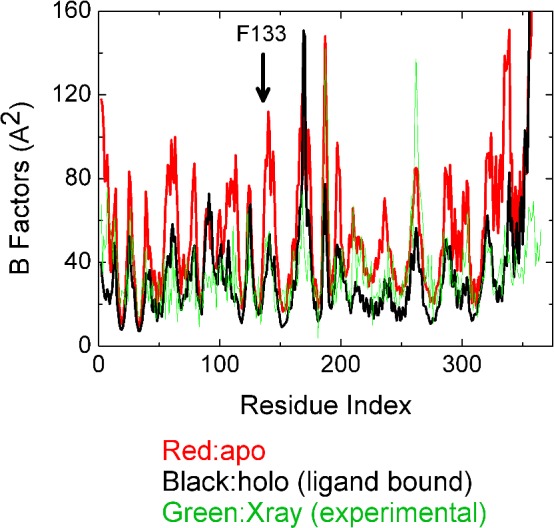
Cyp40 B-factors from apo and holo MD simulations and X-ray Average B-factors are greater for the apo form of the protein for both the cyclophilin and the TPR domains. There is a significant reduction fluctuation in both the TPR and the cyclophilin domains when MEEVD is bound. Phe^133^ forms the base of the active site.

The calculated B-factors determined from the holo and apo simulations are shown in Figure 7. For the backbone atoms of the cyclophilin domain (residues 2–186), the average holo and apo B-values are 31.0 and 50.0 Å^2^ respectively. The C-terminal TPR domain (residues 213–360) is found to be slightly more flexible with B values of 32.6 and 56.2 Å^2^ for the apo and holo forms respectively. It is clear that binding of the MEEVD peptide to the TPR domain has a significant effect on reducing fluctuations on both the TPR and the cyclophilin domains.

The average crystallographic B-factors measured at 100 K were taken from X-ray structure 1IHG. For the backbone atoms of the cyclophilin domain (residues 2–186), the average B-value is 10.9 Å^2^ and for the TPR domain (residues 213–360), the average B-value is 12.5 Å^2^. When these values are converted into corresponding B-values at 310 K (using a scale factor of 310/100) the B values are 33.8 and 38.8 Å^2^ respectively. Figure 7 compares B-factors for the X-ray structure and from MD simulations of the apo and holo models. The simulation results are in good agreement with experiment in predicting regions of low and high fluctuations; however, the apo simulations overestimate the peaks. This probably explains the large average apo B-values.

**Figure 8:**
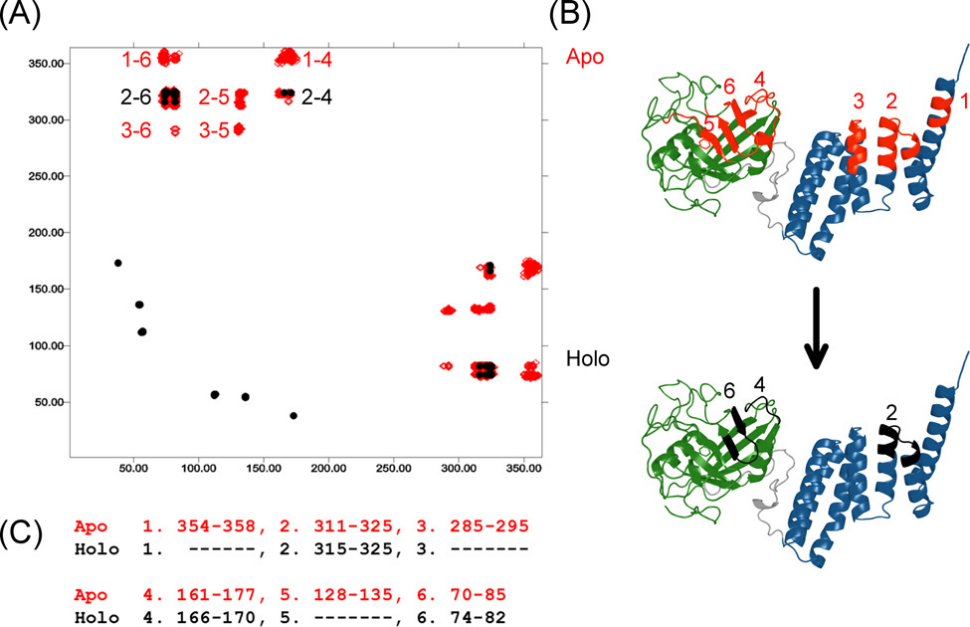
Anti-correlated motions of Apo and Holo Cyp40 (**A**) Anti-correlated motions in Cyp40. MD simulations show clear anti-correlated motions in apo Cyp40 between the cyclophilin and TPR domains (red; regions 1–6). Anti-correlated motions are much reduced on binding to–MEEVD (black; regions 2, 4 and 6). The axes of the cross-correlation matrix graph represent amino acids from the N-terminus. (**B**) Regions showing anti-correlations have been mapped to the structure of Cyp40. (**C**) Specific amino acids for each anti-correlated region.

The measure of correlation between the fluctuations Δ*R*_i_ and Δ*R*_j_ of ith and jth (α-carbon) atoms can be assessed by calculating the projection of the fluctuation of one atom on the other [calculated as the magnitude of the dot product (Δ*R*_i_ · Δ*R*_j_) at every ps) and averaged over the full 100 ns trajectory (Figure 8). It is customary to normalize the correlations *C*_ij_ of Δ*R*_i_ and Δ*R*_j_ as:

$$C_{\mathrm{ij}}=\frac{\langle \Delta R_{i}\cdot \Delta R_{j}\rangle }{{\langle \Delta {R_{i}}^{2}\rangle }^{1/2}{\langle \Delta {R_{j}}^{2}\rangle }^{1/2}}$$

Here, the dot in the numerator denotes the scalar product and the angular brackets are time averages. With this normalization, *C_ij_*varies between–1 and +1. This normalization removes the effect of large fluctuations which would be dominant in the expression (Δ*R*_i_ · Δ*R*_j_).

**Figure 9:**
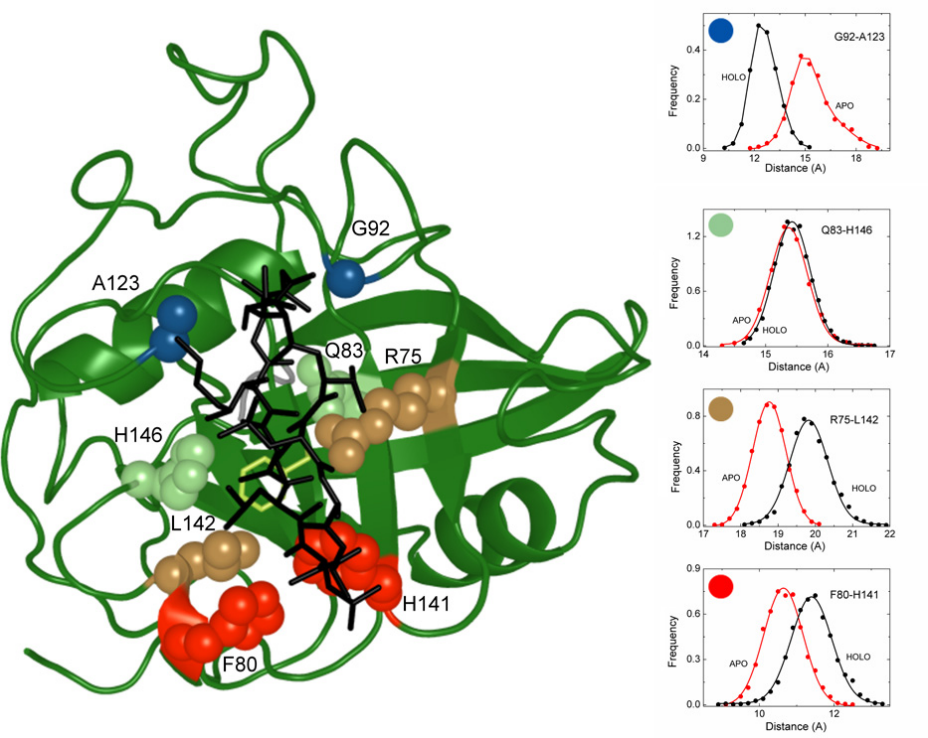
Binding–MEEVD allosterically alters motions in the active site The pair-wise distances between key residues in contact distance with the cyclophilin substrate (or CsA inhibitor) lie on either side of a binding cleft change when MEEVD binds to the TPR domain. CsA is modelled in the active site (1CWA.pdb; black sticks).

The simulation of apo-Cyp40 shows strong anti-correlated motions between the N-terminal cyclophilin domain residues and the C-terminal TPR domain residues The *C*_ij_ values are normalized between +1 and–1 and there are only six regions of the molecule that are involved in significant anti-correlated motions with *C*_ij_ values <–0.75 (Figure 8B). All of the anti-correlation peaks involve interactions between the cyclophilin and TPR domains and there are no major anti-correlated motions within either of the two domains. The six regions are shown in Figure 8; on the TPR domain there is region 1 (residues 354–358 on helix 7), region2 (residues 311–325 on helix 6) and region3 (residues 285–295 on helix 4). Those residues on the cyclophilin domain that are negatively coupled with the TPR regions form a band across the middle of one face of the eight-stranded β-barrel and are composed of three distinct regions: residues 161–177 (labelled region 4) are at the C-terminal end of the cyclophilin domain; region 6 is the pair of antiparallel β-strands comprising residues 70–85 and region 5 is a β-strand (residues 128–135) that lies at the centre of the active site of the cyclophilin domain. It is significant that residues from regions 5 and 6 play an important role in binding ligands at the active site. Figure 9 highlights those residues that make direct interactions with the CsA inhibitor (placed by overlaying the CypA–CsA structure PDB code 1CWA). In particular, Phe^133^ is absolutely conserved in all isomerase-active cyclophilins and is crucial for substrate recognition with its phenyl ring forming the base of the proline-binding pocket.

An analysis of the 100 ns MD trajectories of the holo (MMEVD-bound) Cyp40 complex showed a very significant reduction in the anti-correlated motions between the cyclophilin and the TPR domains. A comparison of the correlation matrices of holo and apo structures (both contoured at *C*_ij_ values less than–0.75) is shown in Figure 8. The only anti-correlations remaining at this level are between regions 2 and 4. The communication between the helices of the TPR domain and the active site has been significantly reduced. The loss of enzyme activity measured on peptide binding may therefore be related to the dampened motions within the catalytic domain and the reduced anti-correlated movements induced by the remote binding of the MEEEVD peptide.

A further analysis of the holo and apo trajectories identifies an interesting distortion of the active site binding cleft in the cyclophilin domain when the remote TPR domain binds the MEEVD peptide. The key residues in contact distance with the cyclophilin substrate (or CsA inhibitor) lie on either side of a binding cleft (Figure 9). The pairwise distances between the Cα atoms of juxtaposed residues on the cleft were monitored and plotted as distance distributions over the course of the 100 ns trajectories. The effect of MEEVD binding to the TPR domain is to increase the distance between Phe^80^ and His^141^ (and R^75^-L^42^) at one end of the cleft while closing the gap between Gly^92^ and Ala^123^ at the other end. The distance between the middle residues (Gln^83^ and His^146^) remains constant. These changes are consistent with a scissor-like motion (Figure 9) that distorts the shape of the binding cleft and may explain the loss of enzyme activity as being due to poorer binding of the substrate.

## DISCUSSION

Previous studies on TPR domains have proposed that plasticity in the TPR domain enable it to adopt a range of conformations with different protein partners [40,41]. The TPR domain of protein phosphatase-5 (PP5) is significantly unfolded at physiological temperatures and binding the–MEEVD C-terminal motif of Hsp90 is thought to be coupled to folding [41]. Using biophysical methods, we have shown that flexibility in the TPR domain of Cyp40 is significantly influenced by temperature and that by forming a complex with–MEEVD peptides the TPR domain of Cyp40 is stabilized. These experimental results were consistent with the MD simulations of the apo and holo Cyp40 structures which showed that binding of the MEEVD peptide to the TPR domain clearly reduces the B-factor for both TPR and cyclophilin domains.

The allosteric effect of peptide binding to reduce PPIase activity of Cyp40 Isomerases by ∼30% was unexpected, but can now be rationalized in terms of protein dynamics. The PPIase mechanism of CypA has been well studied and there is good evidence for a coupling between dynamics and substrate turn-over. NMR relaxation experiments have established that millisecond motions within the PPIase domain of CypA during catalysis occur with a frequency comparable to substrate turnover [5,42–44]. MD simulations have also predicted that there is a coupling between protein dynamics and catalysis [6,45–47]. Extensive mutational studies show it is possible to change the isomerase activity and protein dynamics of CypA by [42], mutating a residue remote from the active site [44,48]. The MD results provided in the present paper suggest an allosteric mechanism in which correlated motion of the TPR domain is required for optimum PPIase turnover through communication between active-site residues on the front face of the β-barrel of Cyp40 that is the supporting template for the active site residues. This communication between the TPR and PPIase domains is dampened by binding to the Hsp90 peptide. The change in shape of the active-site cleft with the scissor-like motion of the substrate-binding residues serves to pinch together at the ‘Abu pocket’ end of the cleft and provides a likely explanation for the poorer substrate binding and enzyme activity. The ‘Abu pocket’ binds to the amino acid Abu in CsA.

The measured dissociation constants between Cyp40 and Hsp90 are relatively weak; for full length Hsp90 at 20°C, the *K*_d_ is 2.9 μM (*K*_d_ for MEEVD is 10.7 μM; Supplementary Figure S2). This suggests that the biological function may require ready dissociation of the Cyp40–Hsp90 complex. The strong temperature dependence of the interaction between Hsp90 and Cyp40 described in the present study is in the physiological temperature range. Upon heat shock, the affinity of Cyp40 for MEEVD is significantly reduced and equilibrium between bound and unbound Cyp40 is shifted towards the more enzymatically-active unbound form of the enzyme. A reasonable hypothesis is that enhanced isomerase activity may be able to rescue partially folded proteins from unfavourable interactions under conditions of heat or possibly other stress conditions.

## Abbreviations

Cyp40: cyclophilin 40
CsA: cyclosporin A
CypA: cyclophilin A
FKBP: FK506 binding protein
Hsp: heat shock protein
ITC: isothermal titration calorimetry
PPIase: peptidyl-prolyl isomerase
RMS: root mean square
TDF: thermal denaturation fluorescence
TPR: tetratricopeptide repeat
wt: wild-type
